# The outdoor time in non-myopic children has decreased to that of myopic children during the SARS-CoV-2 pandemic

**DOI:** 10.22336/rjo.2023.6

**Published:** 2023

**Authors:** Piotr Kanclerz, Carla Lanca, Szymon Adam Radomski, Michał Szymon Nowak

**Affiliations:** * Department of Ophthalmology, Hygeia Clinic, Gdansk, Poland; ** Helsinki Retina Research Group, University of Helsinki, Finland; *** Escola Superior de Tecnologia da Saúde de Lisboa (ESTeSL), Instituto Politécnico de Lisboa, Lisbon, Portugal; **** Comprehensive Health Research Center (CHRC), Escola Nacional de Saúde Pública, Universidade Nova de Lisboa, Lisbon, Portugal; ***** Institute of Optics and Optometry, University of Social Sciences Lodz, Poland; ****** Provisus Eye Clinic, Częstochowa, Poland

**Keywords:** myopia, refractive error, pandemic, outdoor time, screen time

## Abstract

**Objective:** Low levels of outdoor activity are known to be an important risk factor for the development of myopia in schoolchildren. This study aimed to determine outdoor and near work patterns in Polish schoolchildren before and during school closure due to the SARS-CoV-2 pandemic.

**Methods:** All children undergoing a routine pediatric examination in the Elbląg branch of the Hygeia Clinic, together with their parents, were asked to fill an anonymous questionnaire. The subject’s age, spherical equivalent (SE) refractive error, time spent outdoors, screen time and total near work in hours per day before and during the pandemic, were recorded. As substantial differences in physical activity by time of year were reported, activity patterns for June (summer) and December (winter) were recorded. Multiple logistic regression analysis was used to analyze the association between the presence of myopia and outdoor and total near work time at different timepoints.

**Results:** A total of 61 schoolchildren aged 11.95 ± 2.74 (range 7 to 17) years were included in this study. The mean SE in the right eye was -1.78 ± 2.11 with 46% of the individuals (n=28) classified as myopic. Before the pandemic, higher time outdoors was associated with less myopic SE (OR=0.47, 95% Confidence Interval [CI] 0.24 to 0.93). During the pandemic, time outdoors among non-myopic children was similar to myopic children, both during winter and summer months (2.18 ± 1.81 vs. 1.89 ± 1.50; *P*=.51, and 3.47 ± 2.66 vs. 3.31 ± 1.65; *P*=.79 respectively). Time outdoors was not significantly associated with myopia during the pandemic (OR=1.17, 95% CI 0.64 to 2.14). Total near work was not associated with myopia at any time point.

**Conclusion:** The long-term influence of the changing patterns of outdoor and near work on myopia prevalence and progression in our population is still to be established. Nevertheless, it is likely that the decrease of outdoor time may influence the rates of myopia in this region.

## Introduction

Myopia is a common condition that develops in childhood and the prevalence of myopia among children worldwide varies from 0.7% among children aged 3-10 years in Saudi Arabia to 65.5% among teenagers aged 15 years in China [**1**-**3**]. Previously published studies from Poland revealed that the prevalence of myopia varied from 0.4% among children aged 7 years in the city of Poznan to 35.5% among children aged 17 years in Szczecin area, but those studies were performed at the beginning of the twenty-first century [**3**,**4**]. Myopia is an important public health problem, with nearly half of the world population being expected to have it in 2050 [**1**]. Progression to high myopia increases the risk of sight threatening complications in later adulthood, such as myopic macular degeneration. Low levels of outdoor activity are known to be an important risk factor for the development of myopia in schoolchildren [**2**,**5**].

The first cases of the severe acute respiratory syndrome coronavirus 2 (SARS-CoV-2) were reported in China in December 2019, while the global outbreak dates from the beginning of 2020, with the first European lockdowns in February 2020. The SARS-CoV-2 pandemic has led to significant changes in educational patterns, almost half of the world’s students encountering partial or full-time school closure. During school closures, an increase in indoor time and near work activities, such as time spent on digital devices that are potential risk factors for myopia, were registered. Recent studies show an increased myopia prevalence and an accelerated myopic progression during the COVID-19 pandemic lockdowns in children and teenagers of different ethnic backgrounds [**6**-**11**]. However, the exact amount of near work, screen time and outdoor time during lockdown is not known in detail. 

The aim of this study was to determine the outdoor and near work patterns in Polish schoolchildren before and during the pandemic.

## Methods

**Fig. 1:**
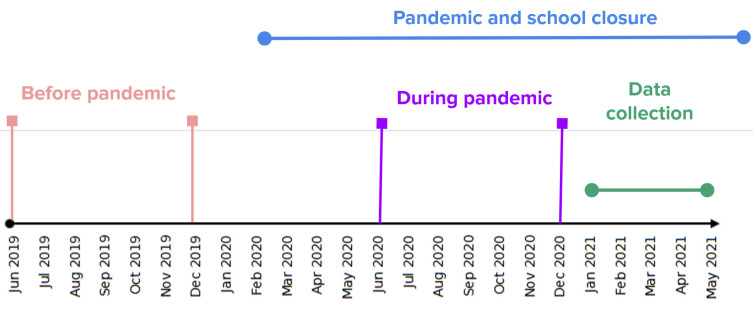
Study timeline. The study was conducted from January to May 2021. The children were asked to fill an anonymous questionnaire regarding their habits in terms of near work and outdoor time before (2019) and during the pandemic (2020). As substantial differences in physical activity by time of year were reported, patterns for June (summer) and December (winter) were recorded

All schoolchildren subscribed to the Elbląg branch of the Hygeia Clinic who underwent a routine medical examination from January to May 2021 were included in the study. Informed consent was obtained from all parents. Children with cataracts, glaucoma, having undergone previous ocular surgery, or suffering from any other ocular diseases were excluded. Together with their parents, the children were asked to fill in an anonymous questionnaire regarding their habits in terms of near work and outdoor time before and during the pandemic. The survey was designed to account for multiple activities and behavioral changes related to weather. Details regarding how many hours a child spent with a computer, phone, tablet, book or notebook and time outdoors were collected. Total near work time was defined as the sum of hours per day spent on the computer, phone, tablet, book and notebook. Outdoor time was defined as the sum of hours per day spent during outdoor activities, walking to and from the school, as well as outdoor activities during classes. As substantial differences in physical activity by time of year were reported, activity patterns for June (summer) and December (winter) were recorded (**Fig. 1**). Two sets of questions were prepared: one for activities before the pandemic and the second regarding their activities during the pandemic. The parents were also asked to state if their children were wearing glasses at the time of the visit and what was their refractive error in these glasses in diopters (sphere, cylinder and axis). Refractive error information was double-checked, if the parents were not certain about the refractive error of the child: (i) they were asked to contact their optician to obtain precise values; (ii) if they have priorly attended to the ophthalmic department of the clinic, these values were filled using prior medical records (iii) if the child was wearing glasses, they were measured with an automatic lensmeter by the nurse (CCQ-800, Yeasn Science & Technology Co. Ltd, Chongqing, China). The questionnaire was considered valid if all the fields, including refractive error, and time spent during the activities across several time points, were filled. The study adhered to the tenets of the Declaration of Helsinki and written consent was obtained for all parents and children provided assent. The study protocol was approved by the local bioethical committee (approval no. KB-37/21).

## Data analysis

Myopia was defined as a myopic refractive spherical equivalent (SE) of 0.50 D or more. Hyperopia was defined as a hyperopic SE greater than +1.0 D. The spherical refractive error (E) was calculated as a sum of the sphere and half of the cylinder. The results were presented as the mean ± standard deviation. The normality of the data was assessed using the Kolmogorov-Smirnov test that showed a normal distribution. In normally distributed data, a paired sample two tailed t-test was performed on compared datasets. Pearson correlation coefficients; values between 0 and 0.3 were considered weak positive, between 0.3 and 0.7 moderate positive, and between 0.7 and 1.0 strong positive linear relationships [**12**]. Multivariate logistic regression analysis was used to analyze the association between outdoor and total near work at different timepoints (before and during the pandemic) with the presence of myopia. A sample size of 54 eyes was estimated to detect a 1-hour difference in outdoor time, based on the estimation of the standard deviation for outdoor time of 2 hours, a power of 95% at a significant level of 5%. A p value less than .05 at a 95% confidence interval was considered as statistically significant. Statistical analysis was performed using Medcalc Software v. 14 (Medcalc Software ba, Ostend, Belgium) and IBM SPSS Statistics v. 28 (IBM Corporation, Armonk, NY, USA).

## Results

The study enrolled 61 schoolchildren, 26 boys (42.6%) and 35 girls (57.4%). All questionnaires were considered valid, and none of the children was excluded from the study. The mean age of included children was 11.95 ± 2.74 years (range 7-17 years). The mean SE in the right eye was -1.78 ± 2.11 D. Twenty-eight children were classified as myopic (45.9%), two as hyperopes (3.3%) and thirty-one as emmetropes (50.8%). The results of the questionnaires are presented in **Table 1**. There was a significant seasonal variation for total near work before the pandemic compared to during the pandemic (*P* =.002 and *P* = .001, respectively), as well as for time outdoors before (2019) and during the pandemic (2020; *P* < .001 and *P* <.001, respectively). More importantly, there was a major change in the total near work during the pandemic, both in summer (*P* < .001) and in winter (*P* < .001). Time outdoors slightly decreased during the pandemic both in summer and in winter, but the difference was not significant (*P* =.38 and *P* = .05, respectively).

**Table 1:** Outdoor time, screen time and near-work in Polish children before and during the SARS-CoV-2 pandemic (n=61)

	Before pandemic (2019)			During pandemic (2020)			Summer difference (before vs. during; *P* value)	Winter difference (before vs. during; *P* value)
	Summer	Winter	*p*	Summer	Winter	*p*		
Time Outdoors	3.51 ± 2.23	2.54 ± 2.65	< .001	3.28 ± 2.25	1.98 ± 1.67	< .001	0.38	.05
Total Near Work	7.50 ± 4.05	8.35 ± 3.84	.002	11.28 ± 4.37	12.37 ± 3.85	.001	< .001	< .001
- Computer or notebook	1.58 ± 1.69	1.98 ± 1.87	.01	5.26 ± 2.68	6.49 ± 2.51	< .001	< .001	< .001
- Cellphone	1.90 ± 2.15	2.31 ± 2.41	.05	3.08 ± 3.04	3.06 ± 2.85	.91	< .001	.002
- Tablet	0.40 ± 1.49	0.26 ± 0.80	.46	0.30 ± 1.11	0.32 ± 1.12	.32	0.66	.68
- Book/ notes	3.61 ± 2.27	3.80 ± 2.25	.32	2.63 ± 2.17	2.51 ± 2.03	.35	< .001	< .001
Time Outdoors	3.51 ± 2.23	2.54 ± 2.65	< .001	3.28 ± 2.25	1.98 ± 1.67	< .001	0.38	.05
All values are presented in hours per day.								

**Fig. 2:**
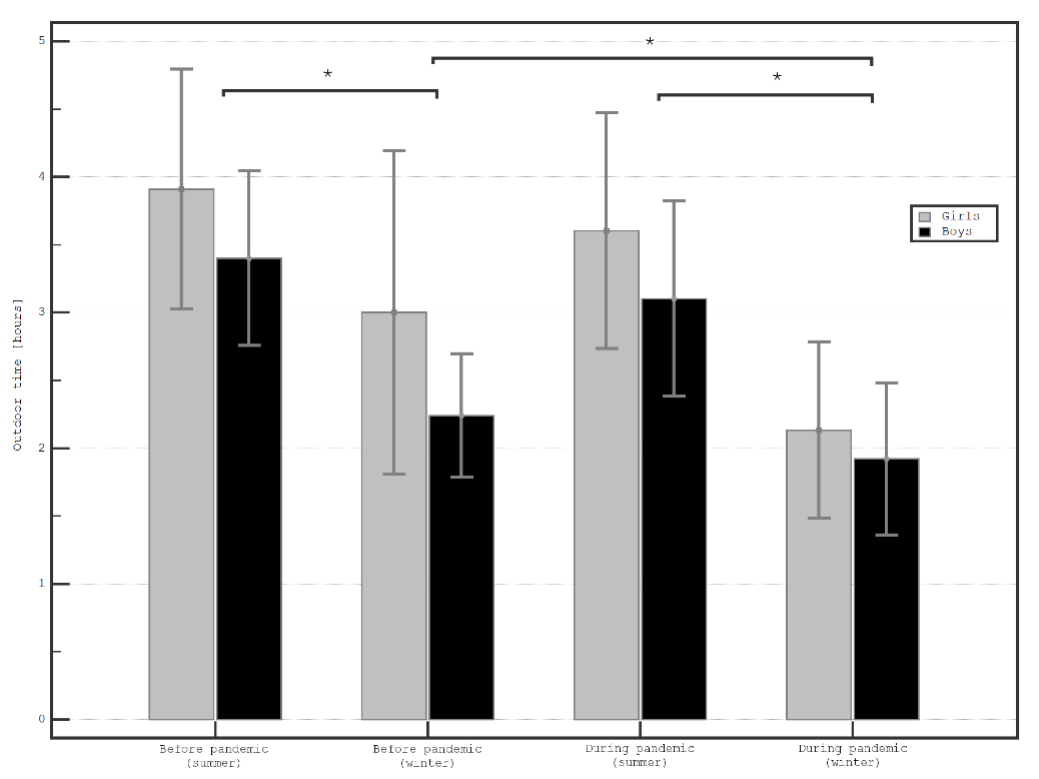
Level of outdoor activity among boys and girls during winter and summer months before, and during the COVID-19 pandemic (n=61)

Girls tended to spend more time outdoors than boys, both before (3.00 ± 3.37 vs. 2.24 ± 1.10; *P* =.39, and 3.91 ± 2.49 vs. 3.40 ± 1.55; *P* =.37 for winter and summer months, respectively) and during the pandemic (2.13 ± 1.86 vs. 1.92 ± 1.36; *P* =.62, and 3.60 ± 2.49 vs. 3.10 ± 1.74; *P* =.39, for winter and summer months, respectively), but the difference was not statistically significant (**Fig. 2**).

**Fig. 3:**
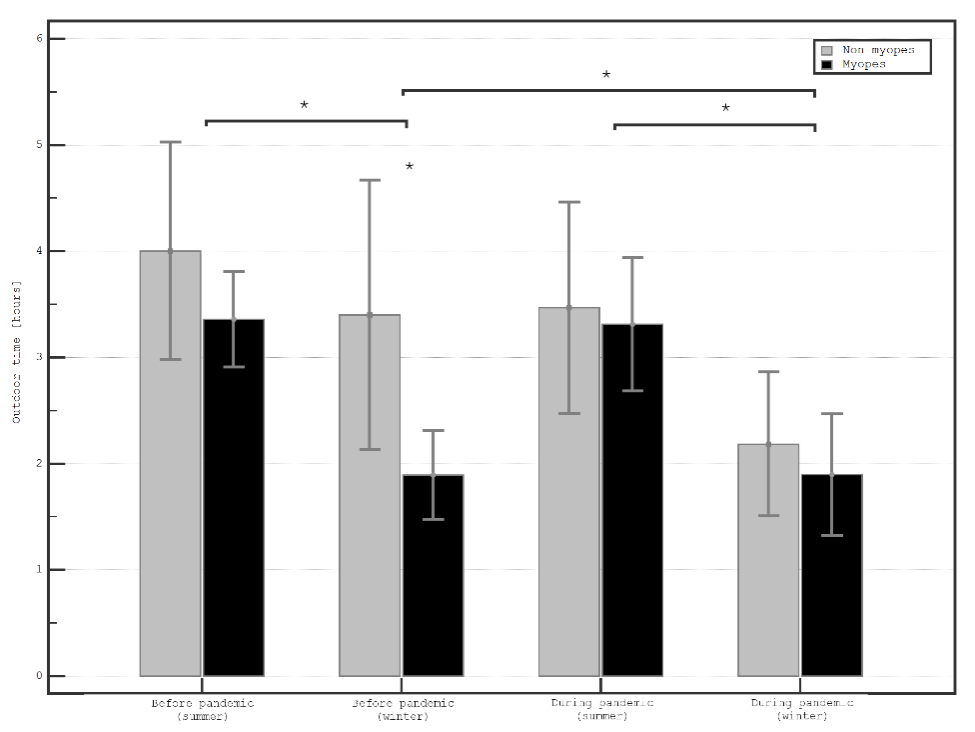
Level of outdoor activity among myopic and non-myopic schoolchildren during winter and summer months before, and during the COVID-19 pandemic (n=61)

The SE of myopic children was -2.70 ± 1.72 D, while for non-myopic children, 0.17 ± 0.45 D. There was no difference in age (11.93 ± 2.75 vs. 11.97 ± 2.76 years, respectively; *P* = .96) and sex (P = 0.70) between myopic and non-myopic children. Before the pandemic, non-myopic children were spending significantly more time outdoors than myopic children during winter months (3.40 ± 3.42 vs. 1.89 ± 1.08; *P* = .03), but the difference was not significant during summer (4.00 ± 2.74 vs. 3.36 ± 1.16; *P* =.26; **Fig. 3**). During the pandemic, the level of outdoor activity among non-myopic children became similar to that of myopic children, both during winter and summer months (2.18 ± 1.81 vs. 1.89 ± 1.50; *P* =.51, and 3.47 ± 2.66 vs. 3.31 ± 1.65; *P* =.79; respectively). There were no statistically significant differences in total near work between myopic and non-myopic children, both before (8.28 ± 3.20 vs. 8.40 ± 4.43; *P* =.91, and 7.08 ± 3.54 vs. 7.91 ± 4.51; *P* =.43 for winter and summer months, respectively) and during the pandemic (11.57 ± 4.08 vs. 13.15 ± 3.52; *P* =.11, and 10.52 ± 4.46 vs. 12.02 ± 4.21; *P* =.18, for winter and summer months, respectively).

The results of the multiple logistic regression analysis are presented in **Table 2**. Higher outdoor time in winter months before the pandemic was associated with lower rate myopia (*P* = .03; OR = 0.47, 95% Confidence Interval [CI] 0.24 to 0.93).

**Table 2:** Multivariate logistic regression analysis of risk factors associated with the presence of myopia at different time points in Polish children before and during the SARS-CoV-2 pandemic (n=61)

Variable	OR (95% CI)	*P*
Total near work [hours/ day] - before pandemic (summer)	0.93 (0.59 to 1.48)	.76
- before pandemic (winter)	1.14 (0.71 to 1.81)	.59
- during pandemic (summer)	1.00 (0.72 to 1.39)	.99
- during pandemic (winter)	0.91 (0.62 to 1.33)	.61
Outdoor time [hours/ day] - before pandemic (summer)	1.37 (0.76 to 2.48)	.30
- before pandemic (winter)	0.47 (0.24 to 0.93)	.03
- during pandemic (summer)	0.96 (0.58 to 1.60)	.87
- during pandemic (winter)	1.17 (0.64 to 2.14)	.61
Dependent variable: Myopia. Abbreviations: OR = odds ratio, CI = confidence interval, SE = standard error		

Half of myopic children spent the least time outdoors before the pandemic in winter (χ² = 3.13, df = 1; *P* = .08), but not in summer (χ2=.02, df=1; *P* =.89) and during the pandemic (χ2 = 1.31, df = 1; *P* =.25, and χ2 = 0.16, df = 1; *P* =.69, for winter, and summer months, respectively). Before the pandemic, there was no correlation between the outdoor time and total near work time (r = .08; p = 0.54, and r = .03; *P* =.81, for winter and summer months, respectively). There was a moderate linear negative correlation between total near work time and outdoor time and during the pandemic summer (r = -0.31; *P* = .02). This correlation was weak in winter months (r = -0.17; *P* =.21) and not significant in multivariate analysis (β =-0.21; *P* =.14).

## Discussion

In this study, we found that before the pandemic myopic children were spending significantly less time outdoors than non-myopic children. This is concordant with what has been previously reported in other countries and the protective effect of time outdoors involves light-stimulated release of dopamine from the retina [**13**]. Importantly, during the pandemic, both myopic and non-myopic children spent similarly little time outdoors. This presumably led to an increase in the prevalence of myopia in that region. We also found that near work increased significantly during the pandemic in all children, and this increase was not different in myopic and non-myopic children. Although near work has been shown to be a risk factor of developing myopia in schoolchildren [**2**], low levels of outdoor activity were shown to be more relevant [**1**,**3**-**5**,**13**,**14**].

Due to the pandemic, schools were partially or fully closed (**Table 3**) from March 2020, in several European countries. However, the level of restrictions in Poland can be considered as one of the greatest in Europe, the schools being closed for 43 weeks, while in Spain only for 13 weeks [**15**]. During the physical closing of schools, remote learning was implemented. The shift in near work habits, associated with the introduction of remote learning, was likely but has not been extensively evaluated. In Polish children included in this study, outdoor and near work patterns changed during the pandemic. Similar restrictions have been applied in the USA. In a recent study including 53 children (8.3 ± 2.4 years) from the USA, electronic device use increased significantly and outdoor time decreased during the pandemic in myopic children (n=14 myopes), but time spent on total near work (including reading and writing) was not significantly different [**16**]. In studies published before the SARS-CoV-2 pandemic, the rates of myopia in European countries have been moderate (**Table 4**) [**2**,**17**]. For example, in a Danish study by Lundberg et al., 17.9% of children (mean age of 15.4 ± 0.7 years) had much lower myopia than in several Asian countries [**17**]. Previous studies have shown that in Australia, European Caucasian children tend to spend more time outdoors than children of East Asian origin [**18**]. However, the children’s pattern of activities become more myopigenic during the pandemic, and particularly associated with the introduction of remote learning. This shift may result in an increase of the prevalence of myopia and higher myopia progression in future.

In this study, it was noticeable that the most significant decrease in time outdoors influenced mainly non-myopic children. During the pandemic, their time outdoors became similar to that of myopic children. A previous study showed that in summer, during the pandemic, children from Houston, USA, spent approximately two hours less outdoors than before the pandemic [**16**]. In our cohort, this difference was much smaller, but still noticeable. Time outdoors is considered one of the major risk factors for myopia. In some countries, outdoor programs have been implemented as public health interventions to curb the myopia epidemic [**19**]. In a recent study, spending time outdoors in childhood was confirmed to be associated with a reduced risk (Odds ratio of 0.82) of myopia in adulthood [**20**]. Additionally, in a recent meta-analysis, spending time outdoors has been also shown to reduce axial elongation (mean difference=-0.03mm) [**21**]. Based on the results of several randomized controlled trials, it is recommended that children should spend at least two hours outdoors every day [**22**-**24**]. The study by Mirhajianmoghadam et al. conducted in the USA, reported similar results to those found in our study; during the COVID-19 pandemic, children demonstrated increased screen time and decreased outdoor activity [**16**]. Nevertheless, associations between near work and myopia have not been consistently observed. A study including children (n=414) aged 7 to 13 years, from the Collaborative Longitudinal Evaluation of Ethnicity and Refractive Error (CLEERE) study, showed that near work was not predictive of risk of juvenile onset myopia [**25**].

In this study, during the pandemic, screen time on smartphones increased nearly by half, while the computer screen time tripled. This was accompanied by a decrease of reading time with books and other non-digital reading materials. A recent meta-analysis has shown that smart device screen time alone (odds ratio 1.26 [95% CI: 1.0–1.6]) or in combination with computer use (odds ratio 1.77 [95% CI: 1.28–2.45]; I2=87%) was associated with myopia in children and young adults [**26**]. Importantly, the viewing distances associated with smart devices is smaller than other forms of near-vision work e.g., reading books, and the duration of exposure is usually longer [**26**]. Moreover, there is a growing body of literature showing that excessive use of digital media is associated not only with myopia, but also psychological, social and neurological adverse consequences [**27**]. The harming role of the change in visual habits should be considered when discussing potential school closures within the following years.

**Table 3:** Total duration of school closure in selected European countries. Table prepared based on findings from Wang J, Li Y, Musch DC et al. [**15**]

Country	Total duration of school closure in weeks*
Bulgaria	41
Finland	33
France	38
Germany	38
Greece	37
Italy	38
Netherlands	31
Norway	29
Poland	43
Portugal	24
Romania	32
Russia	13
Spain	12
Sweden	24
Ukraine	27
United Kingdom	27
* defined as full closure (that refers to situations in which schools where all schools were closed at a nation-wide level), and partial closure (closure of some regions, some grades or reduced in-person instruction), as of when the study was concluded (September 2021).	

**Table 4:** The prevalence of myopia among children in the European countries before the COVID-19 pandemic

Czepita et al. 2017 [**4**]	Poland	SE < -0.5 D	5,601	6-18 years	11.9 ± 3.2	12.2%
Lundberg et al. 2018 [**17**]	Denmark	SE ≤ -0.5 D	307	average age: 9.7, 11.0, 12.9 and 15.4 years (screened at 1-2, 5-year intervals)	15.4 ± 0.7	17.9%
Tideman et al. 2017 [**28**]	Netherlands	SE ≤ -0.5 D	5,711	6 years	6.37 ± 0.7 (myopia) 6.16 ± 0.5 (non-myopia)	2.4%
Matamoros et al. 2015 [**29**]	France	SE ≤ -0.5 D	1,781	0-9 years	N/ A	19.6%
Matamoros et al. 2015 [**29**]	France	SE ≤ -0.5 D	8,289	10-19 years	N/ A	42.7%

Limitations of our study included a small sample size. Additionally, refractive error was reported by parents and only assessed at a single point in time. Other limitations included the questionnaire collection of information about time spent during various activities during the pandemic, only collected at the clinic visit after the pandemic. Validity and reliability of the questionnaire were not tested and potential difficulties with recalling pre-pandemic patterns (almost a year later) may have induced potential biases. Similarly, we did not collect information about various weekday activities, but asked to estimate the average time spent in those activities. Still, one can consider that the behavior patterns did not differ that much between weekdays and weekends, particularly in the winter months, as low outdoor temperatures and precipitation could limit outdoor activities, but not school activities. Furthermore, the cross-sectional design of the study precluded any analysis of how the change in near work and outdoor may affect refractive error in future.

## Conclusions

Outdoor and near work patterns in Polish children included in this study changed during the pandemic. In non-myopic children, before the pandemic outdoor time was higher than in myopic children. During the pandemic, time outdoors decreased and non-myopic children were spending similarly low time outdoors as myopic children. The long-term influence of the changing patterns of outdoor and near work on myopia prevalence and progression in our population is still to be established. Nevertheless, it is likely that the decrease of outdoor time may influence the rates of myopia in this region.

**Conflict of Interest statement**

The authors report no conflicts of interest and have no proprietary interest in any of the materials mentioned in this article.

**Informed Consent and Human and Animal Rights statement**

Informed consent was obtained from all individual participants included in the study.

**Authorization for the use of human subjects**

Ethical approval: All procedures performed in studies involving human participants were in accordance with the ethical standards of the Komisja bioetyczna przy Okręgowej Izbie Lekarskiej w Gdańsku (approval number approval no. KB-37/21) and with the 1964 Helsinki declaration and its later amendments or comparable ethical standards.

**Acknowledgements**

The authors would like to thank Mrs. Katarzyna Rancia from the Hygeia Clinic, for collecting the questionnaires.

**Sources of Funding**

No funding was received for this research. Dr. Kanclerz reports grants from Alcon, non-financial support from Visim and Optopol Technologies, outside the submitted work. Mr. Radomski has nothing to disclose. Dr. Lanca has nothing to disclose.

**Disclosures**

None.
